# Professional and collective identity exploration and coping among mental health practitioners during shared trauma

**DOI:** 10.1186/s40359-026-05056-6

**Published:** 2026-07-07

**Authors:** Lipaz Shamoa-Nir

**Affiliations:** Department of Behavioral Sciences, Zefat Academic College, Safed, Israel

**Keywords:** Professional identity, Collective trauma, Mental health, Resilience, Coping

## Abstract

**Background:**

This study explored the development of professional and collective identities and coping, resilience, and functioning, among mental health practitioners experiencing shared trauma during a period of collective trauma.

**Methods:**

Twenty-four Israeli mental health practitioners (social workers, psychologists, and psychiatrists) who were displaced following the October 7th events participated in semi-structured interviews conducted shortly after evacuation.

**Results:**

The thematic analysis identified four central themes reflecting the role of professional and collective identity in coping and professional functioning under conditions of shared trauma: (a) Professional identity supported emotional containment and ethical decision-making, enabling participants to maintain clinical functioning under conditions of ongoing stress and uncertainty; (b) Collective identity was associated with solidarity, shared meaning-making, and strengthened commitment to clients and colleagues; (c) The two identity dimensions were reported as interdependent and continuously influencing one another, forming a dynamic process rather than separate constructs; and (d) This interaction was described as sustaining coping efforts and facilitating continued professional engagement in the context of shared trauma.

**Conclusions:**

Professional and collective identities appear to function as interconnected processes that support adaptive coping and clinical functioning following exposure to mass trauma.

**Supplementary Information:**

The online version contains supplementary material available at https://doi.org/10.1186/s40359-026-05056-6.

## Introduction

Collective traumatic events impose complex demands on practitioners who are simultaneously members of the affected communities [25]. The October 7, 2023, attacks in Israel exposed civilians to widespread collective trauma and resulted in substantial internal displacement, with an estimated 200,000 Israelis evacuated from their homes [19, 21]. Within this context, clinicians, psychologists, and social workers were often displaced alongside their clients while remaining expected to provide ongoing therapeutic support. Mental health professionals were thus required to deliver care while simultaneously coping with their own exposure to the same traumatic reality, intensifying emotional strain and challenging their professional identity [4]. These circumstances blurred personal and professional boundaries, highlighting their implications for coping, professional identity, and collective belonging under extreme stress.

Recent research on the psychological impact of the Israel-Hamas war has found that mental health practitioners exposed to the same traumatic context as their clients experience a wide range of responses, including anxiety, acute stress, media-induced secondary trauma, and, in some cases, post-traumatic growth and resilience [4]. These findings underscore that shared traumatic reality imposes complex emotional demands on practitioners, who must manage personal distress while continuing to provide therapeutic support during a national state of emergency. Moreover, professional identity has been shown to serve as a stabilizing factor in emotionally intense situations, enabling practitioners to maintain boundaries and uphold ethical standards [17]. The phenomenon of shared traumatic reality, wherein practitioners experience the same disaster as their clients and encounter both personal and professional exposure, has been extensively documented and linked to challenges in emotional regulation and professional boundaries [2, 25]. Such shared exposure can create tensions between professional roles and private life, necessitating unique coping mechanisms among mental health professionals [5, 25].

Identity development, especially in multicultural contexts, has been shown to involve ongoing comparison processes and negotiation of religious, cultural, and gender identities [20]. Professional identity formation has been identified as a dynamic process that influences how practitioners regulate emotions, make ethical decisions, and foster resilience in demanding professional contexts [6, 16, 23]. Research indicates that a coherent professional identity provides practitioners with a cognitive and affective framework that enhances emotional regulation and supports ethical decision-making under stress [6, 16]. Moreover, empirical work across healthcare settings suggests that a well-developed professional identity is associated with greater psychological resilience and adaptive coping, and can mitigate adverse outcomes such as compassion fatigue by strengthening internal resources for managing emotional strain and stress [6, 7].

At the same time, collective identity and shared belonging with affected populations may provide additional psychological resources, such as mutual support, empathy, and enhanced sense of purpose, which can promote both individual coping and solidarity within treatment settings [8]. These shared elements of identity and experience contribute to how mental health practitioners make sense of their work and maintain commitment to care.

Research on post‑traumatic professional growth suggests that exposure to collective trauma can foster new skills, reflective capacities, and adaptive coping strategies among mental health practitioners even amidst ongoing stressors [1, 4]. Studies in related contexts indicate that sustained exposure to shared trauma may present both challenges and opportunities for professional development, shaping therapeutic practice and resilience among mental health practitioners [12, 14].

Existing research has documented the emotional and professional challenges faced by mental health practitioners during mass traumatic events, including exposure to shared trauma and displacement [4, 19, 21]. While prior studies have explored individual coping strategies, professional identity, and resilience, less is known about how these factors intersect with collective identity to shape emotional responses and clinical functioning under extreme stress. Evidence from related fields, such as studies of teachers exposed to mass trauma, suggests that crisis situations can prompt reflection on professional roles, personal values, and career priorities, with experiences of stress or burnout potentially acting as catalysts for professional growth [22]. Building on these findings, this study focuses on displaced clinicians, psychologists, and social workers, exploring how professional and collective identities interact with coping strategies to support functioning, resilience, and adaptive professional development within a context of shared trauma during a period of collective trauma.

## Method

### Participants

Data for this study were drawn from a larger investigation examining the effects of the October 7, 2023, attacks on Israeli citizens, which included 180 voluntary participants. The larger study was designed to examine the psychological consequences of both direct exposure to the October 7th events and continuous exposure to sustained missile attacks across different regions in Israel. Specifically, the broader investigation explored how exposure to the October 7th attacks, alongside ongoing missile attacks in conflict areas near the northern and southern borders, was associated with levels of stress, anxiety, depression, psychological distress, and general health outcomes. The present analysis focused on 24 mental health providers: 13 social workers, 8 clinical psychologists, and 3 psychiatrists (age: M = 30.36, SD = 5.26), including 13 women and 11 men. Regarding marital status, 13 participants were married, 5 were single, and 6 were divorced. Participants reported 3–25 years of professional experience. All were Israeli citizens who were directly exposed to the October 7 Israel-Hamas attacks, including missile threats, and were either personally evacuated or part of a community that temporarily relocated from their homes.

### Procedure

Data were collected in 2024, approximately 7–10 months after the October 7, 2023, Hamas attacks, during an ongoing period of conflict in Israel involving Hezbollah in Lebanon, Hamas, and Iran. Participants were recruited via WhatsApp and Facebook and were provided with a link to register for the study. Informed consent was obtained prior to participation, and participants received a coupon as an incentive. The study protocol was approved by the Ethics Committee (ZAC#A43-2023). All participants were fully informed about the purpose of the study and the use of their data, and they provided consent for their data to be used for research purposes. Participant identities were kept confidential.

### Data collection

Semi-structured, in-depth interviews were conducted 4—8 weeks after evacuation. Each interview lasted 60—90 min and was conducted either via secure video calls (Zoom) or in-person meetings. The interview used in this study was developed specifically for this research. An English-language version has been uploaded as a supplementary file and cited in the main manuscript. Participants were selected to represent varying levels of direct trauma exposure, client populations, and personal characteristics, including parental status and community engagement. The interview guide addressed the following topics:

1. Emotional experiences during displacement and continued professional work.
2. Professional coping strategies and adaptations.
3. Reflections on professional role, ethical decision-making, and professional identity.
4. Perceptions of community solidarity.

### Data analysis

Transcripts were analyzed using thematic analysis [3]. Initial coding focused on identifying experiences, actions, and reflections related to professional and collective identity. Coding was then used to examine relationships between coping strategies, professional functioning, and the formation of collective identity. Credibility was supported through peer debriefing and member checks.

Following Bryman’s (2004) qualitative procedures, the author conducted an initial detailed review of four interviews to identify four preliminary themes. Based on these observations, a coding scheme was developed and applied to all 12 interview transcripts to explore recurring patterns related to professional identity, coping, and well-being. Coding tables were used to document initial codes, theme and sub-theme development, and illustrative examples, while a separate codebook outlined definitions, inclusion/exclusion criteria, and the frequency of coded instances.

To enhance reliability, a research assistant independently performed blind coding, and the resulting codes were compared with the author’s coding, showing high agreement (70%—83%). The analysis presented reflects the first author’s final coding decisions.

## Results

The analysis of interviews with displaced mental health Practitioners revealed a complex and dynamic interplay between professional and collective identity in the context of shared traumatic exposure. Participants described how their dual positioning as both mental health professionals and members of the affected community shaped their emotional experiences, coping strategies, and ethical decision-making processes.

Exposure to collective trauma appeared to both challenge and reinforce professional functioning. Identity processes seemed to play a mediating role in the relationship between exposure and coping, enabling participants to integrate personal distress with ongoing professional responsibility.

### Exposure levels and professional coping

Exposure to ongoing shared trauma emerged as a central contextual condition in participants’ experiences, shaping identity processes, coping efforts, and professional functioning. Within this context, participants described varying levels of exposure to the traumatic events, which shaped both the intensity of their emotional responses and the coping strategies they employed. Exposure was conceptualized along a continuum that included direct exposure (e.g., personal loss, home damage, or physical threat), indirect exposure through clients or colleagues, and combined exposure involving both personal and professional domains. This differentiation highlights a key analytic insight emerging from the data: trauma exposure in helping professions is rarely singular or bounded, but rather multilayered and simultaneously personal and professional.

Participants’ narratives suggest that identity processes functioned as an integrative mechanism that enabled them to hold these multiple layers of exposure together without fragmentation of functioning. As one participant articulated the simultaneity of roles, "I experienced personal loss but was part of saving other families." This dual positioning illustrates how professional identity was not suspended in moments of personal trauma but instead actively constructed the meaning of the experience.

In addition, the findings indicate that exposure level by itself did not determine participants’ coping capacity. Instead, what emerged from the interviews was the way in which exposure intensity interacted with the strength and consolidation of professional identity. Participants who described a more stable and well-integrated professional identity tended to report a greater ability to regulate emotional reactions while still maintaining their professional roles. As one participant explained this dynamic, "Even when the situation was very intense, I felt grounded in my role as a clinician. It didn’t stop the emotions, but it helped me stay focused on what I needed to do." These accounts suggest that professional identity functioned as an internal organizing framework, helping participants structure their experience and sustain functioning under conditions of extreme stress.

Table 1 summarizes the main themes identified in the analysis, highlighting professional coping patterns related to identity dimensions, emotional responses, coping strategies, and identity-related challenges.

**Table 1:** Identity, Coping, and Professional Functioning Following Trauma Exposure

Category	Core Features	Illustrative Quote	Impact on Professional Functioning
Professional Identity	Sense of commitment to the profession, moral anchor, professional knowledge and training	"I remembered I was trained for this; it helped me stay calm."	Provides a framework for action; enables emotional processing while maintaining professional functioning
Collective Identity	Sense of belonging to the community, commitment to the group, shared moral responsibility	"The community gave me strength"; "I did not feel alone."	Enhances solidarity; supports ethical decision-making and peer support
Emotional Responses	Anxiety, sadness, frustration, sense of responsibility	"I was worried but knew I had to continue working with everyone."	Influences capacity to engage adaptive coping strategies
Coping Strategies	Use of professional identity as an anchor, peer support, emotional processing, proactive action	"I discussed challenges with colleagues, and it helped everyone."	Supports flexibility between emotional processing and action; sustains professional functioning
Identity Challenges	Tension between personal and professional identity, boundary management	"It was hard to choose between my family and the team."	Generates ethical dilemmas and stress; requires additional support mechanisms

### Professional and collective identity in shared trauma

A central theme emerging from the analysis is the deep entanglement between professional identity and collective identity. Being part of the affected community intensified emotional experiences, yet it simultaneously reinforced commitment to professional responsibilities. Hence, participants did not separate themselves as professionals from their identity as community members but rather described an increasingly fused identity configuration under conditions of shared trauma. One participant captured this integration clearly: "I felt everything personally, but that made me more determined to be there for everyone, my role as a psychologist became inseparable from my role as a community member that should care for others." This account reflects a process in which collective belonging did not dilute professional boundaries but instead redefined their meaning through shared vulnerability.

A novel insight emerging from this theme is that collective identity did not function only as a social or cultural frame, but also as an emotional regulator that shaped the meaning of professional action. "Moving to the hotel with all our community gave me emotional stability and helped me make sense of the situation and it was clear what I should do professionally in those terrible moments." In this sense, belonging to the affected community appeared to transform professional responsibility into a morally and emotionally anchored position rather than a purely role-based obligation.

### Coping through identity anchoring

Most of the participants consistently described their professional identity as a stabilizing anchor that supported coping during displacement and ongoing exposure to trauma. Participants described how maintaining professional standards, adhering to ethical guidelines, and continuously reflecting on their professional role provided a sense of structure and stability in coping with stress during the traumatic period. As one participant explained, "Everything around me felt uncertain, so in order to keep myself busy I chose to return to my professional role—calling clients, following clinical protocols, and focusing on what I was trained to do, it really helped me organize my thoughts and continue functioning."

Participants described experiences of fear, panic, and emotional distress; however, these reactions did not prevent them from continuing their professional work. Instead, many framed these emotions within their professional role and sense of responsibility, which helped them remain engaged and functional. One participant shared, "When I felt panic, I reminded myself that I was trained for this, and that keeping calm would help the families. Looking back, I guess that awareness kept me functional."

These accounts suggest a unique mechanism than traditional coping models alone: identity anchoring appears to operate as a regulatory system that organizes emotional experience, ethical reasoning, and behavioral action within a coherent professional frame.

### Ethical considerations and collective responsibility

Participants described ongoing ethical tensions stemming from their dual roles as both professionals and community members. These tensions included balancing personal safety, family responsibilities, client needs, and broader community obligations, for example, participants often shared, "It keeps changing all the time" or "it depends on the day and the situation. " As a result, rather than reaching a clear or fixed resolution, participants described these tensions as continuously negotiated within a shifting and unstable traumatic context. They understood these experiences as an ongoing process of balancing competing demands rather than as problems that could be resolved once and for all. One participant explained, "It wasn’t a decision I could solve once. Every day I had to balance things again, depending on what was happening and what was needed at that moment."

In addition, shared community exposure appeared to intensify moral responsibility while also providing a sense of meaning. As one participant reflected, "It was hard to know whether to focus on my own family or the community center. But the community aspect gave me strength since I wasn’t alone in this." This illustrates how collective identity both complicates and supports ethical decision making under conditions of uncertainty.

Ethical reasoning was experienced as both an individual and a socially embedded process. Moral responsibility, in this sense, was understood as something shared and relational that developed through interaction with others, rather than as a purely individual cognitive judgment. One participant reflected on this collective dimension, saying, "I didn’t decide things alone—talking with colleagues and hearing what others were doing helped me to understand what the right thing to do."

### Identity-driven peer support

"Talking with colleagues made me feel part of a team, even in displacement, and we reminded each other why our work matters." This quote illustrates how interactions with peers reinforced both participants professional and collective identity. Through sharing experiences, discussing challenges, and jointly developing coping strategies, they were able to construct meaning from the situation and strengthen group cohesion.

This collective engagement allowed some coping responsibilities to be distributed across the professional group, embedding resilience within a shared framework. As one participant noted, "Talking with colleagues made me feel part of a team, even in displacement. We reminded each other why our work matters." This suggests that resilience in shared trauma contexts is not solely an individual capacity but is also produced relatively through identity reinforcement within professional communities.

### Contextual and individual differences

While the analysis revealed shared experiential patterns across participants, it also highlighted important contextual and individual differences in how identity, coping, and functioning were negotiated. These variations were associated with factors such as professional experience, type of client population, parental status, and level of exposure to traumatic events.

Participants with greater professional experience generally described a higher degree of "flexibility" in integrating their emotional reactions with "diverse professional responsibilities". They often framed emotional distress as manageable within their professional role, drawing on prior experience to maintain a sense of stability and continuity. In contrast, less experienced practitioners more frequently reported feelings of "role confusion", "emotional overload", and "difficulty maintaining clear boundaries between personal and professional domains".

In addition, gender and parental responsibilities appeared to further shape the ways in which participants managed personal and professional identities. In particular, those with caregiving roles described heightened tension in balancing family demands with professional obligations during the crisis period. One participant reflected this complexity, noting, "I was constantly torn between being there for my children and being available for my clients, and I felt there was no clear separation between the two."

### Summary of findings

Across themes, a central insight is that identity functioned as an active regulatory system rather than a static attribute. Professional identity provided structure, continuity, and a sense of procedural and ethical grounding, allowing participants to maintain functioning even under high emotional load. In parallel, collective identity deepened emotional engagement and strengthened moral responsibility, particularly in relation to community needs and shared vulnerability. Together, these findings illustrate that coping in contexts of shared trauma is not solely an individual psychological process, but is embedded within ongoing negotiations between professional roles, collective belonging, and lived exposure.

**Fig. 1:**
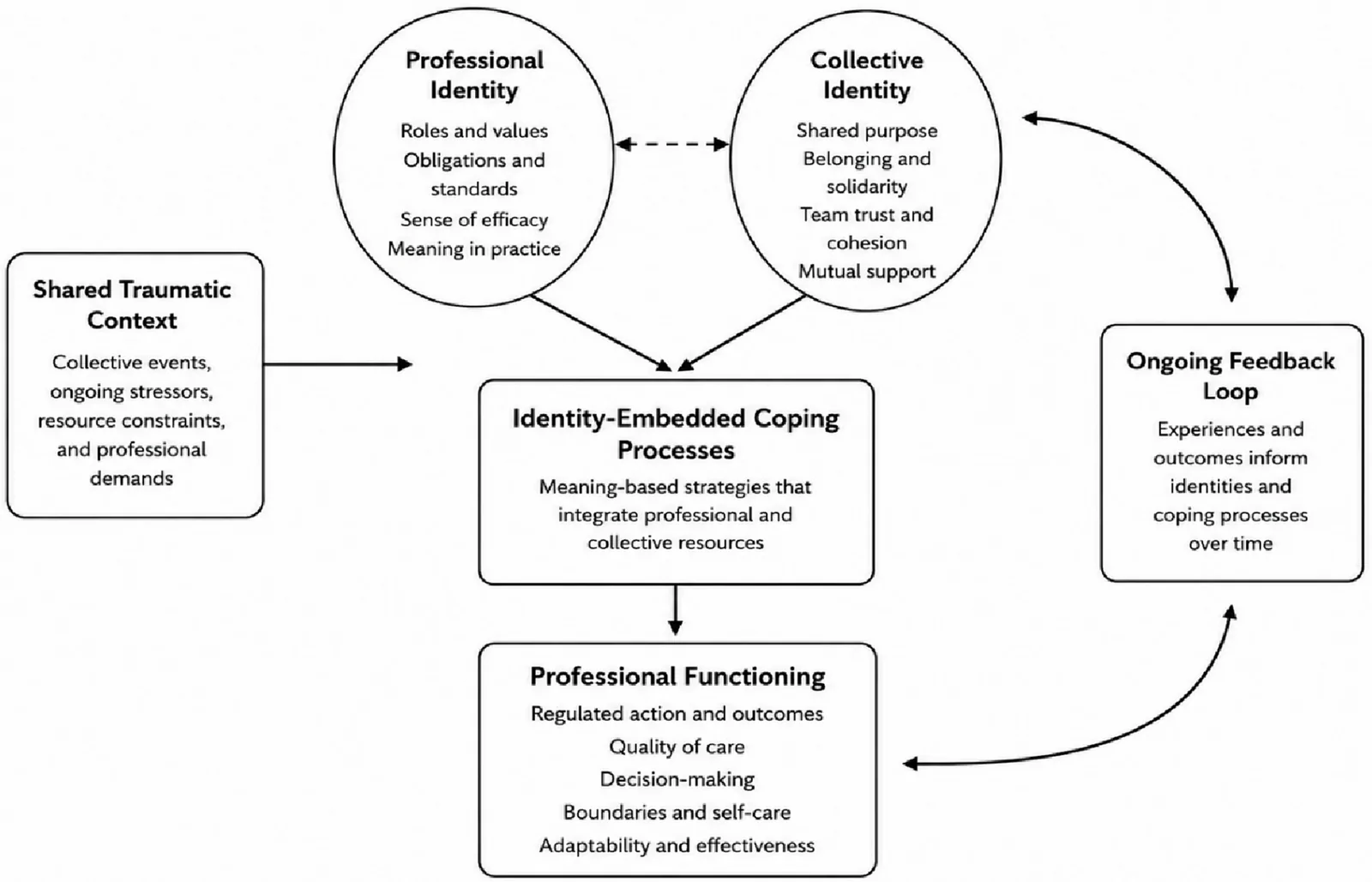
Model of identity-based coping in shared trauma contexts. Note. The model illustrates the dynamic interplay between professional identity and collective identity in shaping identity-embedded coping processes among mental health practitioners exposed to shared trauma. Arrows indicate directional and reciprocal relationships among constructs, highlighting an ongoing feedback loop between coping processes and professional functioning over time

As illustrated in Fig. 1, professional and collective identity processes interact dynamically, shaping coping strategies that help transform emotional experiences into regulated professional functioning.

## Discussion

The October 7, 2023, attacks in Israel created a complex scenario in which mental health practitioners, psychologists, and social workers were displaced alongside their clients while being expected to provide uninterrupted therapeutic support. This shared exposure blurred the boundaries between personal and professional domains, placing mental health practitioners under intense emotional and ethical pressure. The findings of this study underscore the central role of professional and collective identity in sustaining functioning under extreme stress. Consistent with prior research, such circumstances reveal that coping strategies cannot be fully understood without considering the interrelated processes of professional identity and collective belonging [4, 25]. Moreover, these findings align with evidence from multicultural settings highlighting identity negotiation through comparison and integration of multiple social identities [20].

The thematic analysis shows that identity operated in a dynamic way, shaping both emotional responses and coping strategies. In addition, mental health practitioners were able to recognize and acknowledge their own distress while continuing to function effectively in their professional roles, underscoring the integrative role of identity in managing dual exposure to trauma. Within this context, professional identity appeared to function as a stabilizing framework, offering ethical and practical guidelines that supported behavior and helped maintain therapeutic standards [17, 23]. At the same time, collective identity strengthened participants’ sense of commitment to their communities, enabled peer-supported processes of meaning-making, and contributed to resilience through shared responsibility [8].

These findings highlight a dynamic relationship between individual identity processes and collective forms of belonging. Participants reported that being embedded within a community directly affected by terror events appeared to intensify emotional experiences and increase psychological burden. Yet, this shared context was not associated solely with vulnerability. It was also linked to a strengthened sense of ethical engagement, an enhanced perception of moral responsibility, and a deeper articulation of professional purpose [2, 5].

Coping processes emerge as related to ongoing identity development. Participants’ experiences suggest that acknowledging fear, anxiety, or even acute stress reactions did not necessarily compromise their ability to function effectively in their roles. Rather, these emotional responses often coexisted with continued action and sustained professional engagement. This pattern was especially evident when individuals interpreted their experiences through broader narratives of professional mission and communal belonging, which appeared to offer meaning and psychological grounding. In addition, this pattern is consistent with prior research indicating that sustained exposure to collective and shared traumatic contexts may, under certain conditions, contribute to post-traumatic forms of professional growth [1, 22]. Such growth is reflected in developed capacities and the emergence of adaptive coping strategies that evolve over time throughout the course of coping with trauma, within contexts characterized by ongoing stress and continuous exposure to threat [1, 12, 14, 22].

### Practical implications

The findings suggest that strengthening both professional and collective identity may represent a key point of intervention. In this regard, training programs and supervision processes should explicitly address identity-related dynamics as part of professional preparation for situations involving collective trauma [15]. Recognizing collective identity as a psychological resource can help foster solidarity, enhance mutual support, and sustain a sense of professional purpose.

In addition, reflective supervision, peer support, and community-embedded professional frameworks appeared as important contextual resources that support resilience and help maintain coherent professional functioning during prolonged crises [17, 18]. Hence, the development of structured peer networks, alongside supervision frameworks and institutional policies that incorporate identity-informed approaches, may contribute to professional functioning during shared traumatic events.

### Limitations and future research directions

Several limitations should be considered when interpreting the findings of this study. First, data were collected during an ongoing period of conflict, shortly after participants had been displaced due to the October 7, 2023 attacks. This timing may have influenced participants’ responses, reflecting the immediacy of emotional and professional stress rather than longer-term coping, professional growth, or post-traumatic adaptation. Second, while the study focused on professional and collective identity as central mechanisms shaping coping and resilience, other contextual factors, including organizational support, workload, supervision quality, and individual mental health history, were not systematically examined. These factors may interact with identity processes and influence practitioners’ capacity to manage shared trauma. Third, the study focused on specific professional groups (social workers, psychologists, and psychiatrists). The findings reflect the unique experiences of these practitioners within the context of shared trauma and active displacement and may not capture perspectives from other mental health roles or different community and cultural settings, particularly given the heightened emotional and ethical demands of the situation.

Future research could explore the experiences of diverse mental health practitioners in different contexts of collective trauma, examining how professional and collective identities develop over time. Studies might also investigate the influence of organizational factors across various trauma settings could help identify both context-specific and shared processes that support practitioners’ well-being and resilience.

## Conclusion

Taken together, these findings advance an identity-centered understanding of coping in contexts of shared trauma. Rather than conceptualizing coping solely as an individual capacity, the results support a more integrative model in which professional and collective identities function as mutually reinforcing regulatory systems. Within this framework, identity is not a peripheral factor but an active mechanism through which emotional experience, ethical reasoning, and behavioral stability are continuously organized under conditions of prolonged exposure to threat. This aligns with previous conceptualizations of identity as a central regulatory system in stress adaptation [10, 24].

Consistent with research on social identity as a resilience resource in adverse and traumatic contexts [11, 13], professional identity emerged as providing a relatively stable procedural and ethical framework that supports continuity of functioning even under high emotional load. Collective identity, in turn, situates this professional functioning within a broader moral and relational field, thereby strengthening meaning, responsibility, and commitment to action. The interaction between these identity systems appears to facilitate a form of adaptive integration, whereby emotional distress is not eliminated but is instead held and regulated within a coherent sense of purpose and belonging.

From a conceptual perspective, these findings extend prior work demonstrating how group identification enhances perceived personal control, with important implications for health and well-being [9]. This perspective contributes to the trauma and identity literature by suggesting that identity-based regulation plays a central role in shaping emotional experience and professional functioning during shared trauma contexts. Consistent with arguments made in research on shared trauma among clinicians and first responders [2, 5], the study theoretically reframes coping in shared trauma contexts as an identity-mediated process located at the intersection of professional role systems and collective belonging. This shifts the analytical focus from "how individuals cope" to "how identity configurations make coping possible", particularly in contexts marked by continuous exposure and blurred boundaries between personal and professional domains.

## Supplementary Information

Supplementary Material 1. 

## Data Availability

The datasets used and/or analyzed during the current study are available from the corresponding author on reasonable request.
