# Supplementary material for: Professional and collective identity exploration and coping among mental health practitioners during shared trauma
Source: BMC Psychol. 2026 Jul 7;14:1406. doi: 10.1186/s40359-026-05056-6 (PMC13621752; doi:10.1186/s40359-026-05056-6)
Supplement: Supplementary file 1 [file 40359_2026_5056_MOESM1_ESM.docx]

INTERVIEW QUESTIONS

1. Can you tell me about yourself?
2. Background information: gender, age, professional experience, family status, place of residence, and current professional role.
3. Can you describe your emotional experiences during the period of displacement and while continuing your professional work?
4. How have you adapted your professional work to cope with these circumstances? What strategies have helped you maintain your professional functioning?
5. Can you reflect on your professional role during this time? How have your ethical decision-making and professional identity been affected?
6. Can you share examples of situations in which your coping strategies or professional skills were especially important?
7. How do you perceive solidarity and support within your professional or community environment during these challenges?
8. What makes an effective professional team in these circumstances? Can you provide examples?
9. Can you describe a difficult situation you encountered in your professional role and how you managed it?
10. Are there any conflicts or challenges with others (colleagues, clients, or community members) that illustrate your professional coping and decision-making?
11. That is the end of your interview. Is there anything else you would like to add?
12. Do you have any questions for me?
